# Data on correlation between Aβ42 structural aggregation propensity and toxicity in bacteria

**DOI:** 10.1016/j.dib.2016.02.017

**Published:** 2016-02-12

**Authors:** Anita Carija, Susanna Navarro, Salvador Ventura

**Affiliations:** Institut de Biotecnologia i Biomedicina, Departament de Bioquimica i Biologia Molecular, Universitat Autònoma de Barcelona, Bellaterra, 08193 Barcelona, Spain

## Abstract

Protein aggregation and amyloid formation is a hallmark of an increasing number of human disorders. Because protein aggregation is deleterious for the cell physiology and results in a decrease in overall cell fitness, it is thought that natural selection acts to purify aggregating proteins during evolution. This data article contains complementary figures and results related to the research article entitled “Selection against toxic aggregation-prone protein sequences in bacteria” (Navarro et al., 2014) [1]. Here, we used the AGGRESCAN3D (A3D) server, a novel in house predictor that forecasts protein aggregation properties in protein structures to illustrate a striking correlation between the structure-based predictions of aggregation propensities for Alzheimer’s Aβ42 peptide variants and their previously reported deleterious effects in bacteria.

## **Specifications Table**

**Table:** 

Subject area	*Biology*
More specific subject area	*Protein aggregation*
Type of data	*Table, Figures*
How data was acquired	*Aggrescan (bioinf.uab.es*/aggrescan*) and Aggrescan3D (http://biocomp.chem.uw.edu.pl/A3D) predictions.*
Data format	*Analyzed*
Experimental factors	*Aggregation propensities of A*β*42 peptide and two generated mutants F19D and F19D-L34P were analyzed with predictors based on the analysis of the linear sequence and the three dimensional structure.*
Experimental features	*A3D protein predictions are indicated in a table containing the total and average score for A3D prediction, shown as surfaced structures colored according to A3D score and related to biological properties.*
Data source location	*Not applicable*
Data accessibility	*A*β*42 structures* correspond to PDB: 2OTK, PDB: 2BEG, PDB: 2MXU, PDB: 2LMN.

## Value of the data

● The data show that AGGRESCAN3D (A3D) is able to forecast Aβ42 intracellular protein aggregation propensity and its associated toxicity, while allowing visualizing and dissecting the contribution of the regions responsible for this undesired properties in the 3D space.
● The methodology used here to generate data on Aβ42 aggregation properties could be used for the study of the aggregation of other proteins involved in conformational disorders.
● These data are valuable to researchers interested in the relationship between the intrinsic aggregation properties of disease-linked proteins and its associated cytotoxic effect.

## 1 Data

Aβ peptide variants (wild type, F19D and F19D/L34P) aggregation propensities were calculated according to AGGRESCAN [2], [3], which uses protein sequences as input and AGGRESCAN3D (A3D) [4], which instead uses 3D structures. The structures with PDB codes 2OTK, 2BEG, 2MXU, 2LMN, all corresponding to the Alzheimer’s Aβ42 peptide were modeled.

**Fig. 1:**
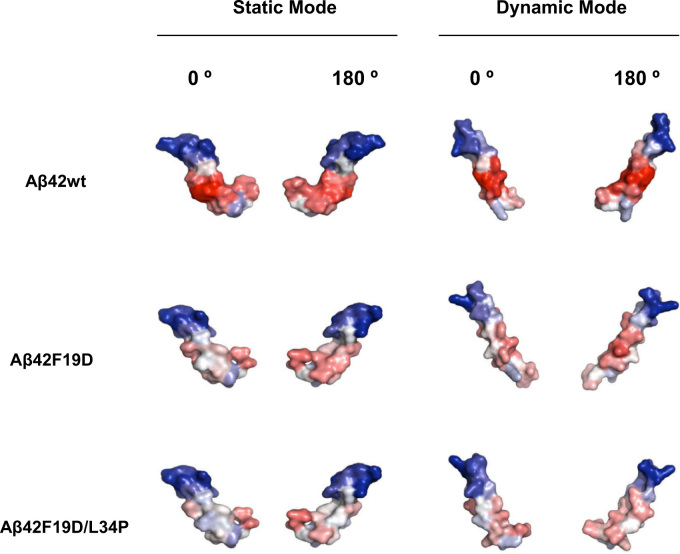
Aβ42wt peptide (PDB: 2OTK:C) and variants F19D, F19D/L34P were modeled and analyzed using A3D in both Static and Dynamic Mode. The protein surfaces shown at 0° and 180° are colored according to A3D score in a gradient from: red (high-predicted aggregation propensity) to white (negligible impact on protein aggregation) to blue (high-predicted solubility). Both Static and Dynamic prediction modes show reduced surface-aggregation propensity in the designed variants when compared with the Aβ42wt.

AGGRESCAN protein aggregation prediction data is provided as the global protein aggregation propensity of the sequence (Na4vSS). With regard to A3D prediction, the total and the average scores corresponding to the overall and average aggregation propensities of the analyzed protein structures are provided. Both in AGGRESCAN and A3D predictions the smallest the score is the highest it is the predicted solubility of the variant (Table 1). The Aβ42 peptide structures corresponding to PDB 2OTK and its variants were modeled using the static and dynamic modes. In Fig. 1 residues are colored according to their Aggrescan3D score. Table 1 and Fig. 1 illustrate the increasing solubilizing effect of the introduced mutations. Because in the used Aβ42 peptide structures the mutated side chains expose to solvent more than 25% of their surface there is a good correlation between AGGRESCAN and A3D scores.

**Fig. 2:**
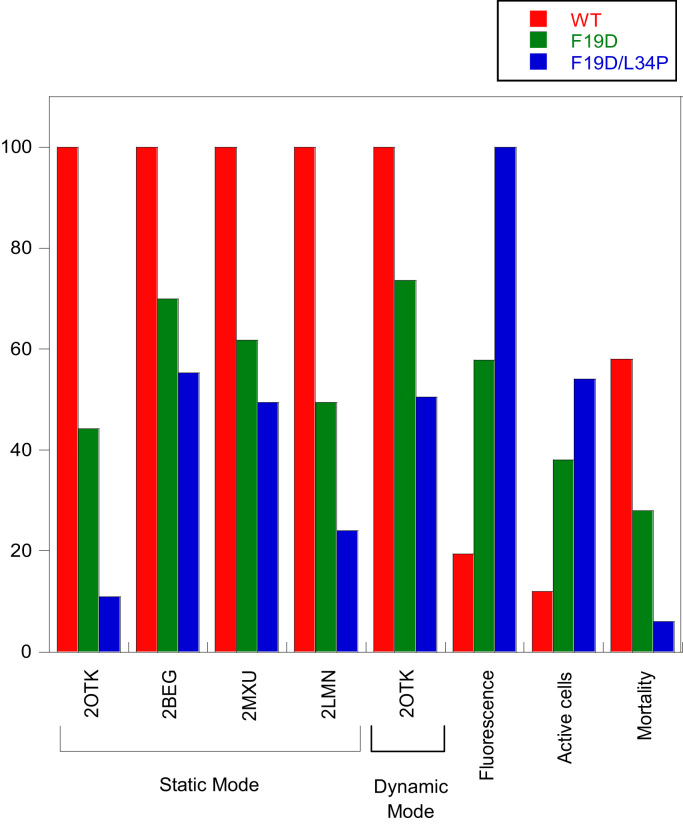
Bar graph comparing the relative predicted aggregation propensities, GFP mean fluorescence as a reporter of protein solubility, metabolic activity and cell viability of variants F19D (green bars) and F19D/L34P (blue bars) with regards to Aβ42wt (red bars). Normalized total scores were obtained by A3D analysis of the indicated PDB files in Static and Dynamic Mode. Aggregation propensities of Aβ42 peptide 3D-structures can be correlated with previous experimental data reflecting the solubility of the protein and their impact in metabolic activity and cellular mortality in the bacterial population.

A3D aggregation propensity data were compared with previously obtained biological data (Fig. 2), observing a striking correlation between the predicted and the experimentally determined solubility, measured as the total intracellular fluorescence of the GFP fused to the specific peptide variant [1]. Not surprisingly, the best correlation with A3D was found for the monomeric 2OTK structure, which in static mode exhibited an *R*^2^=0.994, superior to the correlation found for AGGRESCAN predictions, with *R*^2^=0.960. In the same manner, the A3D predicted aggregation propensity exhibits an excellent correlation with the impact the different peptides have on both cell metabolism and viability [1] (Fig. 2), with *R*^2^=0.998 and *R*^2^=0.999 for the 2OTK structure, respectively; being again more accurate than AGGRESCAN, which predictions exhibit correlation coefficients of *R*^2^=0.978 and *R*^2^=0.988 with the impacts the peptides cause on cell metabolism and viability, respectively.

## 2 Experimental design, materials and methods

### 2.1 Aggregation propensity predictions: AGGRESCAN vs. AGGRESCAN3D

We used two algorithms developed by our group to test their ability to predict the relative aggregation propensities of the Alzheimer’s related Aβ42wt peptide and of two mutants with increased experimental solubility (Aβ42F19D and Aβ42F19D/L34P). AGGRESCAN [2] is a widely used algorithm that employs linear sequence as an input, while AGGRESCAN3D (A3D) [4] is a recently developed algorithm that implements a structure-based approach, uses as input protein 3D-structures derived from X-ray diffraction, solution NMR or modeling approaches and predicts aggregation propensity of initially folded states; this approach resembles that of the previously described Spatial Aggregation Propensity (SAP) suite [5].

Aβ42wt, F19D and F19D/L34P peptide sequences were submitted to AGGRESCAN in FASTA format and Na4vSS (Normalized a4v Sequence Sum for 100 residues) values were selected to compare the predictions. This value is obtained dividing the average aggregation propensity by the number of residues in the input amino acid sequence and multiplying it by 100. Aβ42wt structures corresponding to both the aggregated fibrillar state (PDB files: 2BEG:A, 2MXU:A and 2LMN:A) and the monomeric structure (PDB file: 2OTK:C) were used to analyze the aggregation propensity using A3D. For the fibrils structures the aggregation propensity of a single monomer in the fibrillar conformation was analyzed after energy minimization using the FoldX algorithm [6] integrated in A3D. All PDB files were submitted to A3D in ‘Static Mode’, while only the PDB file (2OTK:C) was also submitted in ‘Dynamic Mode’, since it corresponds to a real monomer in solution and not to a conformer dissected from the fibrillar structure. 10 Ǻ was selected as a distance for aggregation analysis (default sphere radius). The following data were obtained from the output interfaces: average score and total score. The average score allows comparing the solubility of different protein structures. It also allows assessing changes in solubility promoted by amino acid substitutions in a particular protein structure. The total score is a global indicator of the aggregation propensity/solubility of the protein structure. It depends on the protein size. It allows assessing changes in solubility promoted by amino acid substitutions in a particular protein structure as long as they do not result in changes in protein size. The F19D and F19D/L34P mutants were modeled using the FOLDX force field implemented in A3D and analyzed subsequently. Pictures were made using the PyMOL software. The A3D server is available at: http://biocomp.chem.uw.edu.pl/A3D/.

## Figures and Tables

**Table 1:** The aggregation propensity data obtained by AGGRESCAN and AGGRESCAN3D are represented for Aβ42wt peptide and variants F19D, F19D/L34P. Linear sequences were used to obtain Na4vSS (Normalized a4v Sequence Sum for 100 residues) values. To obtain data on the aggregation propensities of 3D-structures, A3D was used in either Static or Dynamic Modes and the indicated PDB files were used as input structures.

Protein	**AGGRESCAN**	**AGGRESCAN3D**									
**Static Mode**								**Dynamic Mode**	
Na4vSS	**2OTK**		**2BEG**		**2MXU**		**2LMN**		**2OTK**	
Average score	Total score	Average score	Total score	Average score	Total score	Average score	Total score	Average score	Total score
Aβ42wt	6.4	0.8	21.0	1.3	33.6	0.8	26.9	0.6	18.0	1.6	40.8
Aβ42F19D	−2.2	0.4	9.3	0.9	23.5	0.5	16.6	0.3	8.9	1.2	30.0
Aβ42F19D/L34P	−6.3	0.1	2.3	0.7	18.6	0.4	11.3	0.1	4.3	0.8	20.6
